# Median Nerve Palsies due to Injections: A Review

**DOI:** 10.7759/cureus.1287

**Published:** 2017-05-29

**Authors:** Andrea Andrea, Jocelyn R Gonzales, Joe Iwanaga, Rod J Oskouian, R. Shane Tubbs

**Affiliations:** 1 Research Division, Seattle Science Foundation; 2 Neurosurgery, Seattle Science Foundation; 3 Seattle Science Foundation; 4 Neurosurgery, Complex Spine, Swedish Neuroscience Institute

**Keywords:** nerve tissue, injections, intramuscular, peripheral nerve injuries, review

## Abstract

Injection nerve palsy (INP) in the median nerve is an iatrogenic peripheral nerve injury that can be inflicted by a faulty intramuscular injection in the median nerve area. The literature reports a 2% incidence of INP among all peripheral nerve injuries. The incidence of INP in developed countries has decreased significantly during the past decade, but the injury appears to remain prevalent in developing countries. A deep understanding of the anatomy of the peripheral nerves, and a precise intramuscular injection technique, have been shown to be vital for preventing INP in the median nerve. Debates continue regarding what, if any, intervention is necessary for injection palsies; and if it is needed, when it should be carried out. In this article, we will review the literature related to median injection nerve palsy and recommended methods of prevention.

## Introduction and background

**Figure 1:**
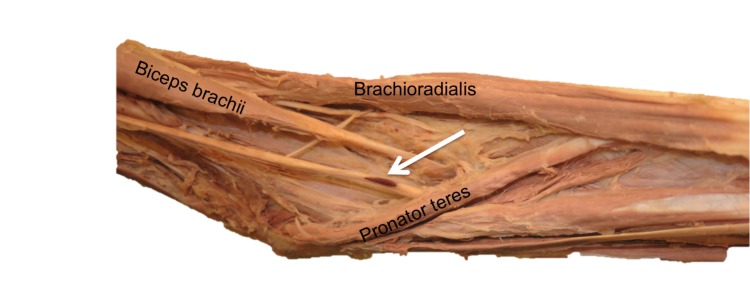
A left-sided hematoma (arrow) in the median nerve within the cubital fossa

**Figure 2:**
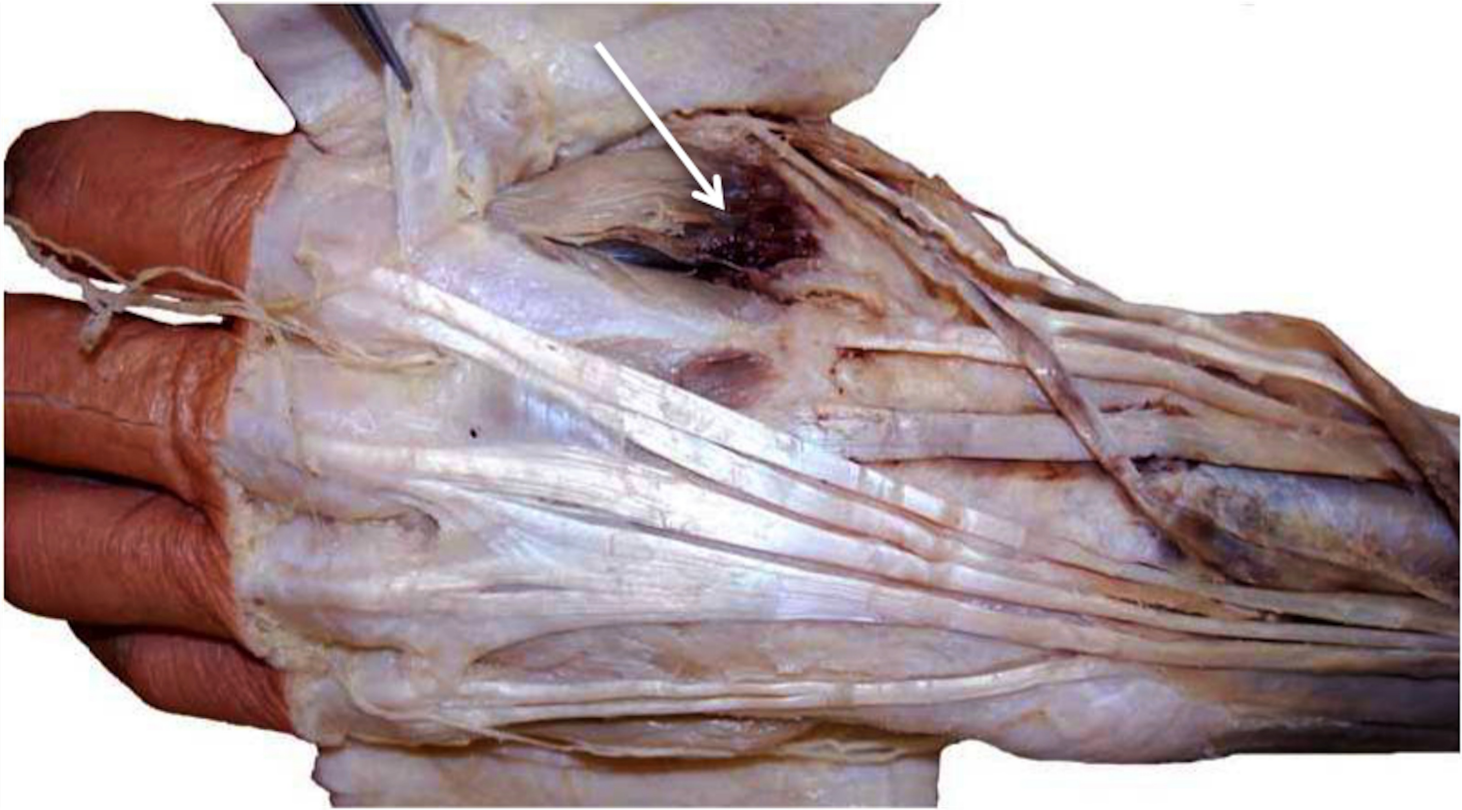
A subcutaneous hematoma following intravenous access. This same specimen was found to have a transection of a branch of the superficial radial nerve as it crossed the anatomical snuffbox

Injection nerve palsy (INP) is an iatrogenic peripheral nerve injury with a reported incidence of 2% among all peripheral nerve injuries recorded (Figures 1-2) [1-2]. The peripheral nerve consists of a variable number of fascicles bound by a loose connective tissue known as the epineurium [3]. Each fascicle is surrounded by the multi-layered perineurium, inside which nerve fibers are packed in a tight bundle [3]. The endoneurium, a thin sheath of connective tissue, surrounds each individual nerve fiber [3]. Under normal conditions, the perineurium acts as a barrier to diffusion (e.g., the blood-nerve barrier) [4-5]. It cooperates with the endothelium of the endoneurial blood vessels to maintain this barrier, which regulates the intrafascicular milieu of the nerve [4-5].

Kalichman, et al. noted axonal and myelin degeneration following the injection of a neurotoxic agent, with a dense concentration of inflammatory cells at the site of nervous injury [6]. Kalichman, et al. speculated that these morphologies indicate nerve injury [6]. Additionally, they emphasized their observation of significant endoneurial edema [6]. In light and electron microscopic studies, edema is characterized as a structureless space in the endoneurium. Under normal conditions, fluid is restricted to the subperineurial region. However, in reported cases of nerve injury, it is present in the perineurial spaces and all parts of the endoneurial compartment of the injured nerves [6].   

Kakati, et al. reported that the onset of symptoms following INP was immediate in 90% of cases, although most patients were not referred until long after their injury [7]. In most cases, patients developed severe pain along the distribution of the affected nerve in addition to paresthesias [7]. The immediate onset of various degrees of motor and sensory deficit was also reported [7]. Motor deficits are generally more severe, while sensory deficits are commonly associated with severe pain, paresthesia, or causalgia along the distribution of the affected nerve [7-8]. The immediate symptoms are most likely to result from the direct effect of injection within a nerve [7-9]. Kakati, et al. suggested perineural fibrosis as a probable cause of the delayed worsening of INP [7].

The anatomical proximity of a nerve to the injection site is the factor that most critically determines the degree of injury [7-8]. The nature and quantity of the injected substance are additional factors thought to contribute to the severity of injury, though to a significantly lesser extent [8]. The drugs reported to be among the most neurologically toxic are: penicillin, diazepam, chlorpromazine, mependine, dimenhydrinate, tetanus toxoid, procaine, and hydrocortisone. The size of the nerve is also thought to correspond to its vulnerability to injury. This explains the high incidence of INP in the large sciatic nerve [7]. Injury to the sciatic nerve has been reported to account for 84.3% of all INP cases, followed by injury to the radial nerve at 5.4% [7].

Western studies have reported a higher incidence of INP in the geriatric population while studies in Southern Asia have revealed a higher incidence in the pediatric population [7, 10-11]. The incidence of INP in developed countries has decreased significantly over the past decade, but the injury appears to remain prevalent in developing countries. Intramuscular injections are often used to treat common illnesses such as fever and infections [10]. Pandian, et al. reported that 86% of injection palsies in North India resulted from improper administration of intramuscular injections by an uncertified medical practitioner [2]. Kotwal, et al. estimated that around half of the injections performed in developing countries are unsafe [12].

In the following section, we will describe injection nerve palsies with regards to the median nerve in more detail.

## Review

### Median nerve injection palsy

Median nerve palsy accounts for 3.6% all reported cases of INP [7]. The median cubital vein, located close to the median nerve, is most commonly used to draw blood and administer IV fluids. It is most commonly caused by inaccurate injections of local anesthetics [8].

In 1990, Linskey and Segal published a case study on a diagnosis of median nerve injection palsy [9]. The injury occurred in a 24-year-old male diagnosed with a left carpal tunnel syndrome. He was injected with 1 ml of methylprednisolone suspended in polyethylene glycol and mixed with 0.5% bupivacaine hydrochloride. The intention was to direct the injection into the patient’s left carpal tunnel. During the injection, the patient experienced “a new type of pain” (i.e., not associated with carpal tunnel syndrome) shooting down his hand into his first two fingers. The symptoms did not diminish after the injection had been completed. The patient was later referred to a neurosurgery clinic where exposure of his left median nerve by sectioning the transverse carpal ligament revealed the presence of an opaque white foreign material within the epineurium. The patient also underwent an epineurectomy, during which a similar white precipitate was observed within several nerve fascicles. Such thickening was previously reported by Posch and Marcotte, who presented an intraoperative picture of a white plaque within the thickened median nerve epineurium [13]. They referred to the plaque as a “median nerve granuloma” [14].

While motor function is more severely affected than sensory function in most cases, the opposite was true for the patient in Linskey and Segal’s case study [9]. This was proposed to reflect the primarily sensory function of the distal median nerve and to indicate that only some nerve fascicles had been affected [9]. Consequently, a spontaneous recovery is highly likely, although a residual deficit would generally remain [9].

Among the substances injected, methylprednisolone has been reported as intermediately toxic [15]. Curtiss and Tucker reported bupivacaine as one of the least toxic local anesthetics, yet claimed that it can nevertheless cause significant axonal degeneration [16]. Chino, et al. and Combes and Clark provided evidence of the neurotoxicity of the polyethylene glycol in which the methylprednisolone was suspended [17]. Linskey and Segal compared their observations with past studies in which the steroid was the sole injected substance in order to determine the main cause of the patient’s injection palsy [9, 16]. Since a similar white plaque was present in these cases, it was deduced that methylprednisolone was the toxin responsible for the patient’s neurological impairments [9].

Another case of median nerve injection palsy was reported by Fremling and Mackinnon in 1996 [8]. The patient was a 68-year-old female who developed an abscess on the medial aspect of her mid-upper left arm following a cat scratch. An incision intended to drain the abscess was performed by her family physician. The skin was initially anesthetized with 1% lidocaine and persistent discomfort was experienced following the injection. During a second injection, the patient complained that she had lost sensation in her thumb, index, and long fingers, and that she was no longer able to flex her thumb and index finger. The patient later failed to regain her sensory and motor functions along the distribution of her left median nerve [8]. Two days after the incident, surgical exploration revealed that the median nerve was intact. Despite the absence of overt indications of nerve injury, the patient continued to experience severe pains. A series of medications including amitriptyline, clonidine, and desipramine were prescribed over the following six months. Nevertheless, her pain remained severe. Nerve conduction studies around five months after the cat scratch indicated a severe median nerve injury. There was no response for median sensory palmar latency, although normal distal motor latency was retained. After seven months, the patient experienced severe hyperalgesia. Her evaluation revealed the loss of functional sensory and motor function along the distribution of her left median nerve [8]. On the basis of her experience of severe chronic pain, a surgical procedure with nerve grafting was performed. The patient noted no change in her motor function postoperatively. During a follow-up 18 months post-surgery, only partial relief of pain was reported [8].

### Clinical relevance/application

A deep understanding of the anatomy of the peripheral nerves, and a precise intramuscular injection technique, have been shown to be vital for preventing injection nerve palsy. Severe radiating pain should indicate an injection directed inside a nerve [8-9]. It is critical the patient experiencing such pain should immediately notify their provider and the injection immediately stopped. Additional precautions should also be taken since more potentially neurotoxic agents are involved. A heightened risk for injection palsy is often associated with patients who are heavily sedated (i.e., under regional or general anesthesia), as they are unable to notify their providers of uncommon symptoms, such as extraordinary pain during injection [8-9].

The experimental work by Kalichman, et al. established previously unreported clinical facts regarding injection-induced nerve palsy [6]. They noted the neurotoxicity of a wide spectrum of lipophilic anesthetics, rather than just a few specific agents. Furthermore, while it had traditionally been believed that only intraneural injections can inflict significant nerve injury, Kalichman et al. discovered that substantial injury can be induced by the extraneural injection of local anesthetics [6]. This finding further supported the hypothesis that the endoneurial blood-nerve barrier is disrupted by neurotoxic agents in the perineurial sheath. It was proposed that alteration in permeability allows an injected neurotoxic agent to affect the nerve directly [10].  

For steroids, Linskey and Segal suggested an ulnar injection to the palmaris longus tendon [9]. If no palmaris longus is present, the injection should be made in line with the fourth digit [9]. It is important to pay close attention when positioning the needle tip [9]. It is advised the needle tip should lie beneath the transverse carpal ligament but outside the median nerve epineurium [9]. The importance of a patient’s subjective response during the procedure cannot be stressed enough. The injection is to be stopped immediately if there is any indication of pain or paresthesia [7-9]. Debates continue regarding what, if any, intervention is necessary for injection palsies; and, if it is needed, when it should be carried out. Kakati, et al. suggest that surgical treatments can help if performed within a reasonable period following the injury [7].  A three to six-month observation period is recommended to determine whether there is spontaneous recovery [9]. This can then be used to decide whether further intervention is necessary [9].

## Conclusions

Complications following intravenous and intramuscular injections are relatively low; however, nerve injury after such procedures can occur. Therefore, the clinician should be aware of this possibility and have a heightened sense of the regional anatomy around the target injection site. In this paper, we introduced the topic of injection nerve palsy and reviewed the literature regarding the incidence rate of symptoms and possible treatments. Nerve palsy due to injection is a serious complication due to the reported symptoms, and an open "error culture" instead of a "blame culture" must be promoted.
